# Arylated gold nanoparticles have no effect on the adipogenic differentiation of MG-63 cells nor regulate any key signaling pathway during the differentiation

**DOI:** 10.1186/s13104-021-05594-9

**Published:** 2021-05-19

**Authors:** Muhammad Abdulwahab, Amir Ali Khan, Sallam Hasan Abdallah, Muhammad Nasir Khan Khattak, Bizuneh Workie, Mohamed Mehdi Chehimi, Ahmed Ali Mohamed

**Affiliations:** 1 Department of Applied Biology, College of Sciences, University of Sharjah, Sharjah, 27272 UAE; 2 Human Genetics and Stem Cells Research Group, Research Institute of Sciences and Engineering, University of Sharjah, Sharjah, 27272 UAE; 3 Department of Chemistry, Delaware State University, 1200 North DuPont Highway, Dover, DE 19901 USA; 4 Univ Paris Est Creteil, CNRS, ICMPE, UMR7182, 94320 Thiais, France; 5 Department of Chemistry, College of Sciences, University of Sharjah, Sharjah, 27272 UAE

**Keywords:** Gold-aryl nanoparticles, Stem cells, Adipogenic differentiation, Signaling pathways

## Abstract

**Objective:**

MG-63 cells that have osteoblastic and adipogenic differentiation potential were evaluated for internalization, and adipogenic differentiation in the presence and absence of the covalently functionalized aryl gold nanoparticles (AuNPs-C_6_H_4_-4-COOH).

**Results:**

Inductively coupled plasma, flow cytometry and confocal microscopy analyses confirmed that gold nanoparticles were easily internalized by MG-63 cells. The MG-63 cells were differentiated into adipocytes without gold-aryl nanoparticles and with the gold-aryl nanoparticles at 5 µM concentration in both induction and maintenance media. The lipid content assay and the relative expressions of PPAR-γ, ADR1, GLUT1 and GLUT4 genes showed no significant variation with and without the gold nanoparticles treatment. Differential phosphorylation levels of 43 kinases phosphorylation sites were evaluated using the human phospho-kinase array to assess the effect of the gold nanoparticles on the signaling pathways during the differentiation. No kinase phosphorylation site was differentially phosphorylated with two or more folds after the nanoparticles treatment after the first day as well as at the end of MG-63 cells differentiation. The gold-aryl nanoparticles do not affect MG-63 cells differentiation into adipocytes neither do they affect any key signaling pathway. These properties make these gold nanoparticles suitable for future drug delivery and medical applications.

**Supplementary Information:**

The online version contains supplementary material available at 10.1186/s13104-021-05594-9.

## Introduction

MG-63 cells are osteosarcoma cells but they have the same differentiation potential as Mesenchymal Stem cells (MSCs). They are the cancer form of osteoblasts. By differentiation, these sarcoma cells regain the early state of MSCs characteristics and for that reason, they may be differentiated into cells of mesodermal origin such as osteoblasts and adipocytes [1, 2]. Studies have shown that MG-63 cells may be differentiated into adipocytes [3, 4].

Recently, we synthesized robust covalently functionalized gold nanoparticles attached through aryl carbon with the structure AuNPs-C_6_H_4_-4-COOH (abbreviated as AuNPs-COOH) by the reduction of aryldiazonium-AuCl_4_ salts [5]. These gold-aryl nanoparticles are robust under different pH conditions. They have shown outstanding biocompatibility. Besides, they are not prone to aggregation under several physiological conditions. Another important fact related to this interesting class of gold nanoparticles is the ability to modify the organic shell to synthesize highly water-soluble bioconjugates which can maintain small sizes in solution [6, 7]. They have shown outstanding biocompatibility and unique applications in DNA stabilization, Bovine Serum Albumin (BSA) delivery, cellular uptake, lysozyme bioconjugation for antibiotic-resistant bacteria and insulin defibrillation [6, 7]. Furthermore, our recent study also shows that these aryl-gold nanoparticles having various protein coats are easily uptaken by MG-63 cells [8]. Unlike the gold nanoparticles that are reported to have citrate shells, gold-aryl nanoparticles have aryl functional groups covalently linked through carbon to the gold core and highly water dispersible which make them ideal for cellular uptake study [5]. A few classes of nanoparticles have been evaluated in stem cells differentiation such as silica nanoparticles and magnetic iron nanoparticles [9, 10]**.** Size, shell and type may affect the route of stem cells differentiation [9–12]. Hence, nanoparticles have great therapeutic potential for regenerative medicine [13, 14]

The effect of gold nanoparticles on Mesenchymal Stem Cells (MSCs) differentiation has been reported; however, only one study reported their effect on osteoblastic differentiation of MG-63 cells [15]. Conjugated gold nanoparticles also affect cellular proliferation and differentiation. Since our gold-aryl nanoparticles are unique, we aim to assess the effect of gold-aryl nanoparticles on the adipogenic differentiation of MG-63 cells. Two signaling pathways, mitogen-activated protein (MAP) kinase and p38 kinase, were reported to be regulated by gold nanoparticles during osteoblastic and adipogenic differentiation of MSCs [16, 17]. Our study has focused on screening the regulation of various signaling pathways that may be regulated due to the presence of our unique gold-aryl nanoparticles. A human phospho-kinase array was used to assess the effects of the gold-aryl nanoparticles on the phosphorylation levels of 43 kinases phosphorylation sites. These 43 sites consists of kinases or their protein substrates and they are the components of several key signaling pathways and we aimed to find out which pathways are modulated by the uptake of the gold-aryl nanoparticles by MG-63 cells [18]. To the best of our knowledge, this is the first study that screens several signaling pathways in the presence of covalently functionalized gold nanoparticles during the adipogenic differentiation of MG-63 cells. Any gold nanoparticles that do not affect the cellular processes such as signaling pathways can be used for drug delivery and lineage tracing experiments during embryonic development. However, prior to the application of these aryl-gold nanoparticles in biomedicine, further research is needed to unravel the effects of these nanoparticles in other human cells.

## Main text

### Methods

#### Nanoparticles synthesis

The gold nanoparticles were synthesised as previously reported [5]. Briefly, [HOOC-4-C_6_H_4_N≡N]AuCl_4_ (0.031 g, 0.064 mmol) was dissolved in 30 mL of DI water and (0.270 mL, 0.135 mmol) of 9-borabicyclo(3.3.1)nonane (9-BBN) was added dropwise to the salt solution. The reaction was vigorously stirred for 2 h. The nanoparticles formed were purified using a 30 cm length of dialysis tubing cellulose membrane of 25 mm width and AuNPs-COOH stock solution was stored in the refrigerator. 1 mL of AuNPs stock solution was centrifuged at 12,000 g. The nanoparticles pellets were washed with PBS and resuspended in 1 ml of PBS before their use in the differentiation.

#### Internalization of the aryl gold nanoparticles by MG-63 Cells

Some nanoparticles were tagged with FITC [8]. Afterwards, MG-63 cells were treated with the tagged gold nanoparticles for 24 h. Flow cytometry and confocal microscopy methods were used to confirm the uptakes of the nanoparticles [8].

MG-63 cells were treated with gold-aryl nanoparticles with 5, 10, 20 and 40 µM concentrations for 24 h. Afterwards, the nanoparticles were removed, and the cells were lysed to assess the gold concentration by Inductively coupled plasma spectroscopy (ICP).

#### MG-63 cell differentiation into adipocytes

To assess the effects of the nanoparticles, the protocol for the differentiation was slightly modified from our previous studies [2, 19]. Briefly, adipogenic differentiation was induced by induction medium I (DMI) containing a combination of 0.5 µM/mL of 3-isobutyl-1-methylxanthine (IBMX) and 0.1 µM/mL Indomethacin, 1 µM/mL insulin (INS), 0.25 µM/mL dexamethasone, 0.1 µM/mL Indomethacin with 5 µM/mL of the gold-aryl nanoparticles in the treated group and without gold-aryl nanoparticles in the control group. After 48 h, the media were changed to differentiation maintenance media II (DMII) containing 1 µg/mL insulin (INS) with 5 µM/mL gold-aryl nanoparticles in the treated group and without nanoparticles in the control group for 48 h. This two-steps adipogenic differentiation protocol was repeated for 3 cycles with a total of 12 days. The derived adipocytes were stained with Oil Red O and Nile Red [19].

#### Lipid content quantification

Lipid quantification was calculated from the three replicas of treated and control adipocytes [19]. Briefly, Oil Red O stain was eluted with 500 µL of 100% isopropanol at room temperature. Then, 100 μL of the samples were quantified by ELISA at 500 nm absorbance (O.D.) for treated and control adipocytes.

#### Gene expression study via RT-PCR

Real-time gene expression was performed [19] to assess the effects of the nanoparticles on adipogenic regulatory genes. The primers sequences for the genes are given in the additional file 1. Qiagen rotor gene thermal cycle was used to perform real-time PCR using Go Taq qPCR Master Mix. The results were analyzed using Qiagen rotor gene qPCR software and delta-delta Ct calculation method [20].

### Proteome profiler human phospho-kinase array

Adipocytes cells at the end of differentiation as well as cells after 24 h of differentiation with and without the treatment of nanoparticles were subjected to lysis buffer provided by the manufacturer of the array kit (ARY003B, Minneapolis, MN 55413, USA) [21, 22]. Protein concentrations were calculated using Bio-Rad DC Protein Assay kit following manufacturer instructions. A solution of 2.0 mL of diluted cell lysate was incubated on the array overnight at 2–8 °C, followed by washing with Phosphate-buffered saline (PBS) buffer to remove extra proteins. A 1.0 mL of diluted Detection Antibody Cocktail A and B, for part A and B membranes, were incubated for 2 h at room temperature followed by three times washing to remove unbound and extra antibodies on the membrane. One mL of diluted Streptavidin- HRP was added into each well containing membrane for 30 min. Enhanced chemiluminescent detection of the protein blots was photographed using the Bio-Rad Gel Doc system. Quantification and analysis were performed with ImageJ 1.52a software.

### Statistical analysis

All experiments were performed in triplicates and results expressed as the means ± standard deviation of the independent triplicate assays. Statistical significance was analyzed using two-tailed T-test considering significance at p-value < 0.05.

## Results and discussion

### Assessing the internalization of gold nanoparticles

**Fig. 1:**
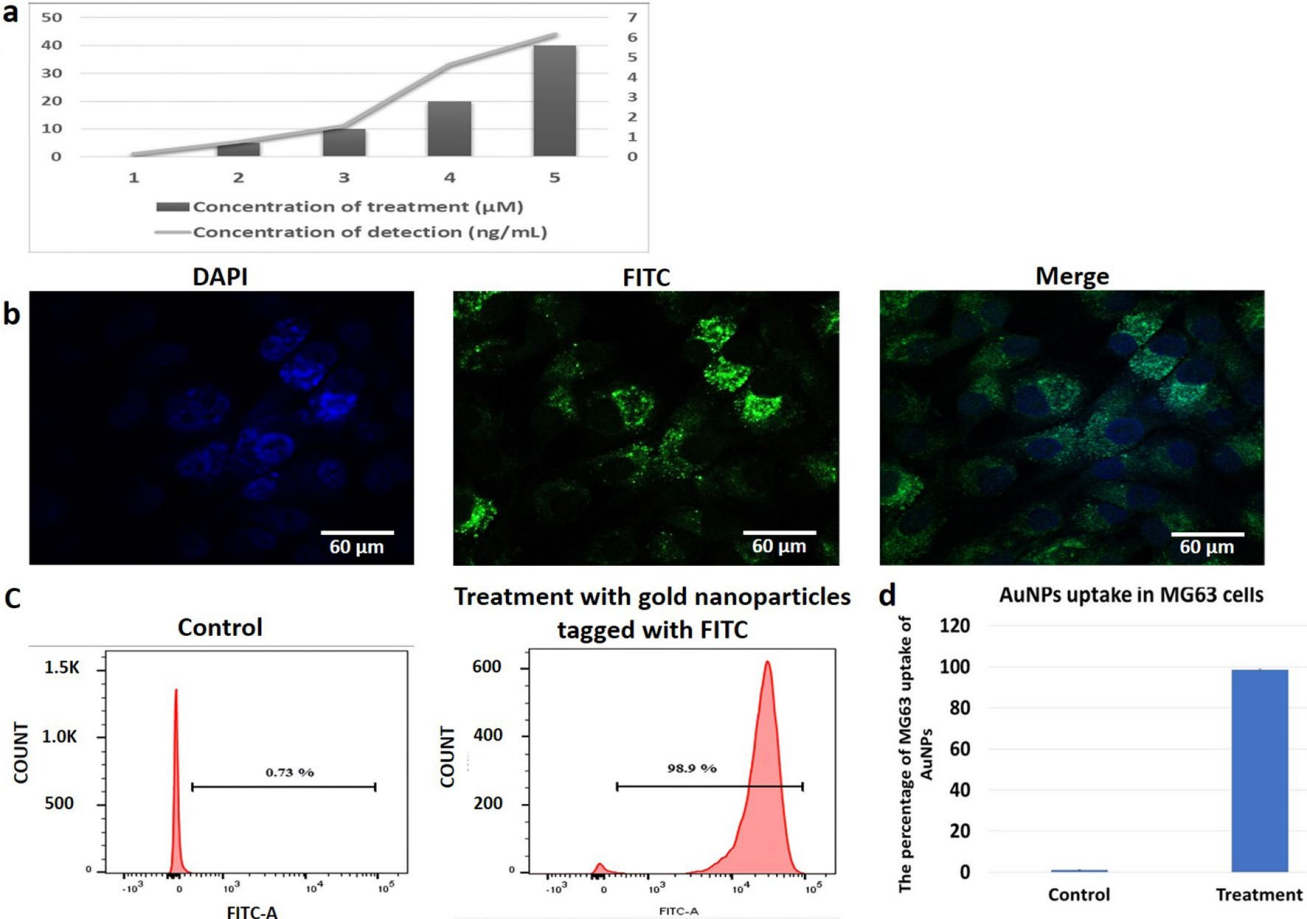
**a** ICP Spectroscopy of the gold-aryl nanoparticles. As the treatment concentration of the nanoparticles increases so does the concentrations of gold per mL of the lysate indicating the MG-63 cells uptake the gold-aryl nanoparticles. **b** Confocal image of a representative MG-63 showing the internalization of FITC-conjugated nanoparticles. Nucleus is stained with DPI. **c** Representative histograms of FITC-conjugated gold-aryl nanoparticles internalized by MG-63 cells. **d** The treated cells had high emission and percentage of cells that internalized the gold nanoparticles

To assess the uptake of gold-aryl nanoparticles by MG**-**63 cells, the cells were treated with 5, 10, 20 and 40 μM concentrations for 24 h. As the treatment concentration increased, so was the concentration of Au detected per mL of the cell lysate (Fig. 1a).

Gold-aryl nanoparticles were also conjugated with FITC and then they were dissolved in PBS with 5 μM concentration. Confocal microscopy and flow cytometry results indicate that FITC conjugated gold nanoparticles were easily internalized by MG**-**63 cells (Figs. 1b, c, d). The treated cells had higher fluorescence emission and the confocal microscope images show the gold-aryl nanoparticles accumulated inside MG-63 cells.

### Evaluation of the differentiation of MG-63 cells into adipocytes with and without the presence of aryl-gold nanoparticles

**Fig. 2:**
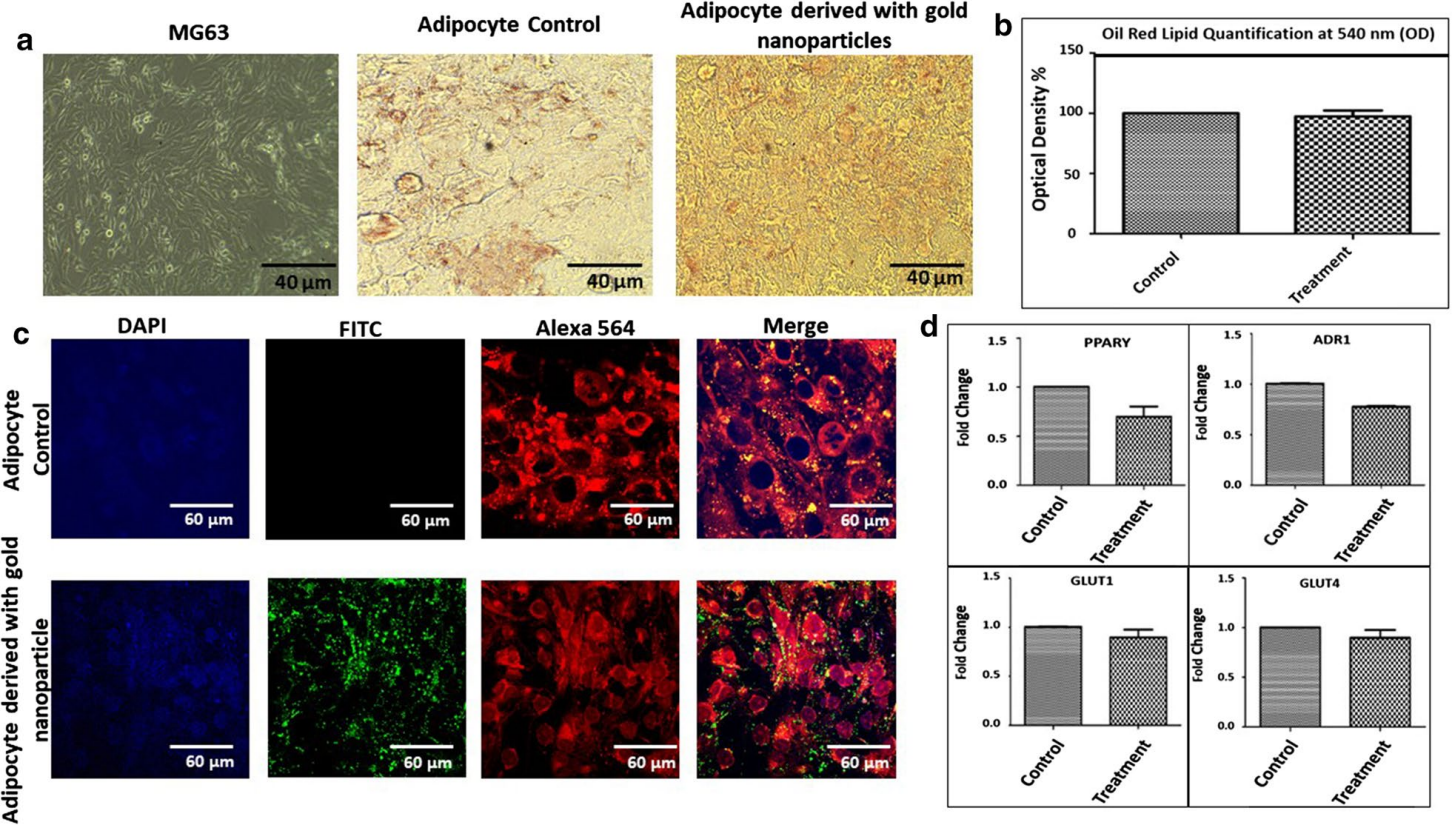
**a** Representative image of MG-63 cells, adipocytes differentiated with and without the gold nanoparticles treatment. **b** Lipid content assay of three biological replica of differentiation of MG-63 into adipocytes. The lipid assay shows no significant difference between treated and control samples. **c** Representative images of adipocytes differentiated with and without nanoparticles treatment. Nuclei were stained with DAPI. Adipocytes were stained with Nile red staining. Confocal images of the adipocytes differentiated with and without FITC-conjugated gold nanoparticles. **d** Real-time PCR of the 5 adipogenic markers. There was no statistically significant variation in the relative expression of these markers in the presence of gold nanoparticles

The nanoparticles at 5 μM concentration were present in both induction and maintenance media of the three cycles. Differentiation was also carried out without nanoparticles (control). Figure 2a displays representative images of the differentiation. The differentiation was evaluated by lipid content assay which shows that adipogenesis was not affected by the presence of the gold-aryl nanoparticles (Fig. 2b). Differentiation was also carried out with the gold nanoparticles conjugated with FITC to observe and confirm the presence of nanoparticles during the entire differentiation process (Fig. 2c).

We also evaluated the adipogenic differentiation of MG**-**63 by the relative expression of specific markers: PPAR-γ, ADR1, GLUT1 and GLUT4 (Fig. 2d). There was no statistically significant differential regulation among these markers in the presence of gold nanoparticles nor was any significant variation in the lipid content.

### Evaluation of phosphorylation level of the 43 kinases phosphorylation sites in the presence and absence of aryl-gold nanoparticles

**Fig. 3:**
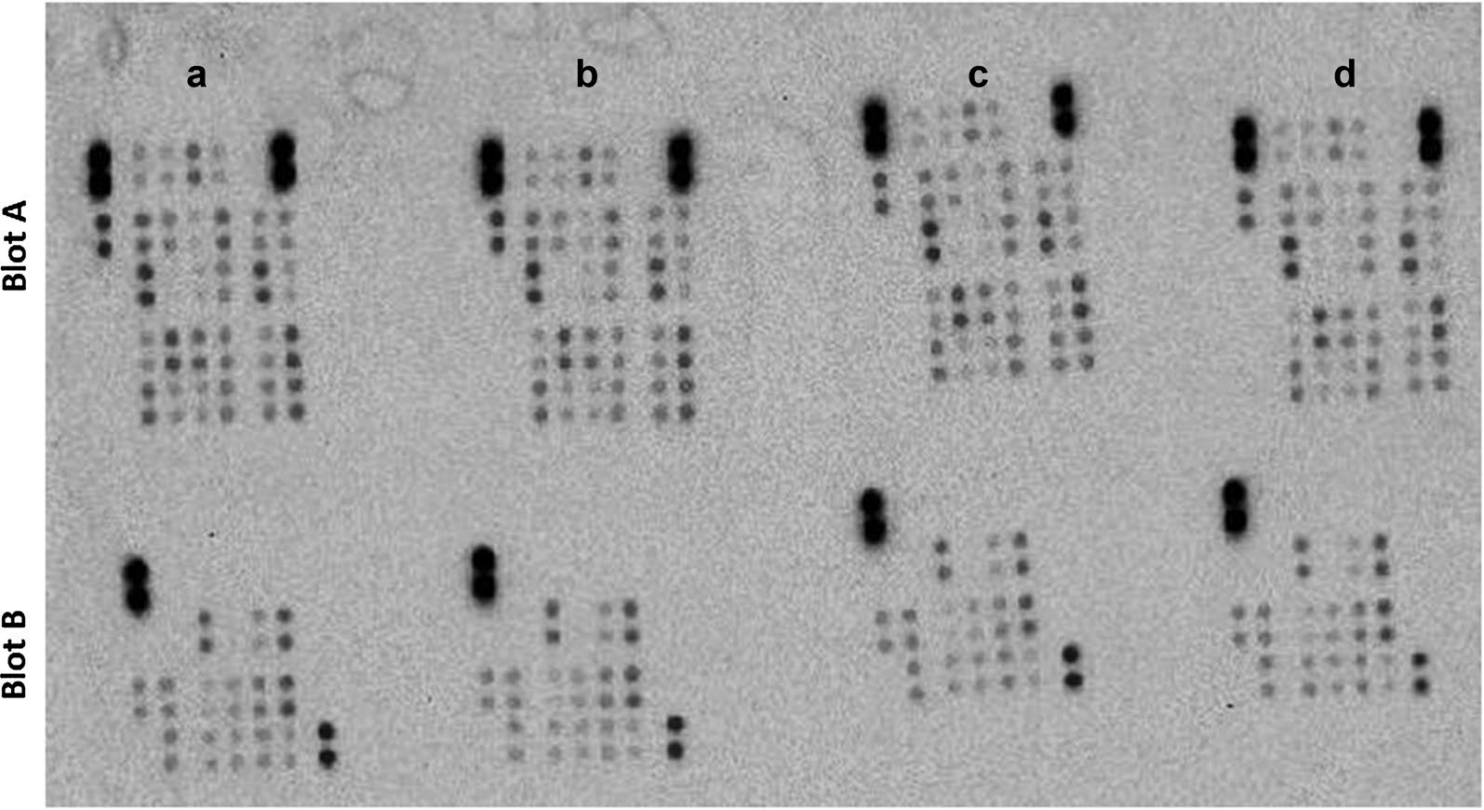
**a** and **b** Phospho-protein array was performed on lysate after 24 h treatment with gold nanoparticles compared with control (**b**) during the first day of differentiation. **c** and **d**: Phospho-protein array was performed on lysate after end of the differentiation in the presence and absence of gold nanoparticles (**d**). No phosphorylation site was differentially phosphorylated two or more folds

The differential phosphorylation level was studied for different kinases or their protein substrates which are the components of the key signaling pathways. We analyzed the phosphorylation levels of these 43 kinases phosphorylation sites; some of them are in the kinases and other are on their protein substrates. The analysis was carried out by using the phospho**-**kinase array during the adipogenic differentiation. In this array, selected antibodies against specific phosphoproteins (sites of kinases) were spotted on nitrocellulose [23, 24]. After cells lysates were incubated on the array, a mixture of biotinylated antibodies against the phosphoproteins were applied followed by Streptavidin conjugated with horseradish peroxidase. The array analysis shows that gold-aryl nanoparticles did not regulate any of the major signaling pathways at the two-point interval, 24 h and after 12 days, during the differentiation as the phosphorylation level of none of the sites varied for two or more folds (Fig. 3). The two-fold changes were considered significant variation [25, 26].

It can be concluded that no kinase or their protein substrates were differentially phosphorylated with the presence of gold nanoparticles. We considered the upregulation and down regulation with two or more folds as significant. Any difference in phosphorylation level is due to the change in chemical and biological environment induced by the addition of gold nanoparticles. This in turn determines the behavior of cells. Analysis of phosphorylation levels of the 43 kinases or their substrates at the end of differentiation indicates that no kinase or their phosphorylation of substrates was differentially regulated at the end of differentiation.

Nanoparticles that regulate signaling pathways will affect cellular behavior and will affect their physiology and biochemistry [27]. Our gold nanoparticles do not have any effects on the cellular signaling pathways and can be used as drug delivery vehicle to cells. Based on this study, these unique gold nanoparticles have great therapeutic application in the treatment of Osteosarcoma and future work should be focused to unravel this application [28]. Furthermore, these gold-aryl nanoparticles may also be used in lineage tracing experiments during animal development.

## Conclusions

The present study indicated that gold-aryl nanoparticles do not affect the adipogenic differentiation of MG**-**63 cells. Furthermore, no key signaling pathway was affected by the uptake of the nanoparticles by MG-63 cells, providing a support that they may be ideal for drug delivery and tracing experiment during development. Hence, these gold-aryl nanoparticles can potentially be useful for drug delivery in Osteosarcoma patients, as they do not affect the cellular processes in these cancer cells. The cancerous cells can be potentially targeted with gold nanoparticles conjugated with drugs that would kill cancer cells. Further studies should also be done to assess the effects of these nanoparticles in MSC differentiation as well as the osteoblastic differentiation of the MG-63 cells. To use these specific nanoparticles for wider medical applications, further research is warranted to unravel the effects of these nanoparticles on other human cells.

## Limitation of the study

The study did not evaluate the effects of aryl-gold nanoparticles on the differentiation of MG-63 cells into osteoblasts.

## Supplementary Information

**Additional file 1**: **Table S1**. qPCR primer sequences of the marker genes.

## Data Availability

All data generated and analyzed during this study will be freely available to any scientist wishing to use them for non-commercial purposes upon reasonable request from the corresponding authors.

## Abbreviations

MSC: Mesenchymal stem cells
RT-PCR: Real Time quantitative PCR
