# Supplementary material for: Arylated gold nanoparticles have no effect on the adipogenic differentiation of MG-63 cells nor regulate any key signaling pathway during the differentiation
Source: BMC Res Notes. 2021 May 19;14:192. doi: 10.1186/s13104-021-05594-9 (PMC8132363; doi:10.1186/s13104-021-05594-9)
Supplement: Supplementary file 1 — Additional file 1: Table S1. qPCR primer sequences of the marker genes. [file 13104_2021_5594_MOESM1_ESM.docx]

**Table 1**. qPCR primer sequences of the marker genes

| **Gene** | **Forward** | **Reverse** |
| --- | --- | --- |
| **GAPDH** | 5′-AGGGCTGCTTTTAACTCTGGT-3′ | 5′- CCCCACTTGATTTTGGAGGGA-3′ |
| **GLUT-4** | 5′-CTTCATCATTGGCATGGGTTT-3′ | 5′-AGGACCGCAAATAGAAGGAAGA-3′ |
| **GLUT-1** | 5′-ATACTCATGACCATCGCGCTAG-3′ | 5′-AAAGAAGGCCACAAAGCCAAAG-3′ |
| **ADR-1** | 5′-TTCTTCCTCATGGCTGTGATGT-3′ | 5′-AAGAAGCGCTCAGGAATTCG-3′ |
| **PPR-Ɣ** | 5′-TTCTCCTATTGACCCAGAAAGC-3′ | 5′-CTCCACTTTGATTGCACTTTGG-3′ |
